# Recurrent Pyogenic Cholangitis: Disease Characteristics and Patterns of Recurrence

**DOI:** 10.1155/2013/536081

**Published:** 2013-05-25

**Authors:** Ye Xin Koh, Adrian Kah Heng Chiow, Aik Yong Chok, Lip Seng Lee, Siong San Tan, Salleh Ibrahim

**Affiliations:** Hepatopancreaticobiliary Service, Department of General Surgery, Changi General Hospital, Singapore 529889

## Abstract

Recurrent pyogenic cholangitis (RPC) is characterized by repeated infections of the biliary system with the formation of stones and strictures. The management aims are to treat acute cholangitis, clear the biliary ductal debris and calculi, and eliminate predisposing factors of bile stasis. Operative options include hepatectomy and biliary drainage procedures or a combination of both; nonoperative options include endoscopic retrograde cholangiopancreatography (ERCP) or percutaneous transhepatic cholangiography (PTC) guided procedures. This current study compares the operative and the nonoperative management outcomes in patients with RPC in 80 consecutive patients. In addition, we aim to evaluate our approach to the management of RPC over the past decade, according to the various degrees of severity and extent of the disease, and identify the patterns of recurrence in this complex clinical condition. Initial failure rate in terms of residual stone of operative compared with nonoperative treatment was 10.2% versus 32.3% (*P* = 0.020). Long-term failure rate for operative compared with non-operative treatment was 20.4% versus 61.3% (*P* = 0.010). Based on multivariate logistic regression, the only significant factors associated with failure were bilaterality of disease (OR: 8.101, *P* = 0.007) and nonoperative treatment (OR: 26.843, *P* = 0.001). The median time to failure of the operative group was 48 months as compared to 20 months in the nonoperative group (*P* < 0.010). Thus operative treatment is a durable option in long-term resolution of disease. Hepatectomy is the preferred option to prevent recurrent disease. However, biliary drainage procedures are also an effective treatment option. The utility of nonoperative treatment can achieve a reasonable duration of disease free interval with minimal complications, albeit inferior to operative management.

## 1. Introduction

Recurrent pyogenic cholangitis (RPC) was first recognized as a distinct clinical entity in the 1930s by Digby [1]. He recognized that intrahepatic biliary calculus occurred more frequently in Hong Kong Chinese as compared to Europeans. In the 1950s, Cook proposed the subsequent term “recurrent pyogenic cholangitis” to describe the condition as a repeated infection of the biliary system with the formation of biliary stones and strictures in the biliary system [2]. Repeated infections of the biliary system have been implicated as one of the important causes of disease progression resulting in complications like biliary cirrhosis and cholangiocarcinoma [3, 4]. 

The therapeutic goals for the management of recurrent pyogenic cholangitis should include firstly amelioration of the acute biliary sepsis by decompression of the infected biliary tree and this can be accomplished nonoperatively by percutaneous transhepatic biliary drainage or endoscopic sphincterotomy and/or biliary stent placement, secondly, complete clearance of calculi and particulate debris from the biliary tract, via methods such as percutaneous transhepatic cholangioscopic lithotomy (PTCSL) and peroral cholangioscopy and thirdly, and the elimination of bile stasis which is essential for preventing recurrent cholangitis and stone formation. Therefore, it is imperative that the anatomical abnormalities including biliary tract ectasia and strictures are addressed and rectified definitively if possible. There is a myriad of interventions, including radiologically guided and endoscopic procedures, as well as surgical procedures including operative biliary drainage and hepatectomy [5–10].

However, the clinical presentation and subsequent management of RPC vary significantly, depending on the location and extent of the calculi, strictures, or abscesses in the biliary tree. Furthermore, the different management approaches are limited by the state of the patient's comorbid conditions and preexisting liver function. Due to the complexity of the treatment and the inherent propensity for stone recurrence, there is yet to be a consensus to the optimal approach.

The objective of this study is to retrospectively analyze the disease profile of the patients treated at a single Asian tertiary institution. In addition, we aim to evaluate our approach to the management of RPC over the past decade, according to the various degrees of severity and extent of the disease, and identify the patterns of recurrence in this complex clinical condition. 

## 2. Materials and Methods

This is a Singhealth Centralised Ethics Board approved retrospective review of a prospectively maintained database of patients with recurrent pyogenic cholangitis at Change General Hospital Singapore, an acute tertiary centre. Between first January, 2002 and October 1, 2011, 89 consecutive patients were diagnosed with RPC. Eighty patients subsequently underwent treatment by the hepatopancreaticobiliary service at our institution; nine patients defaulted before the initiation of treatment and were excluded from the study. 

## 3. Diagnostic Criteria

The diagnosis of RPC was made based on the following premises: firstly, the presence of cholangitis defined by clinical pictures suggestive of cholangitis, including but not limited to the symptoms of fever, jaundice, right upper quadrant or central abdominal pain and this was supported by a biochemical picture of raised white cell count (WBC), bilirubin (Bil), and alkaline phosphatase (ALP), consistent with cholangitis, secondly, imaging investigations suggestive of RPC evidenced by the presence of intrahepatic stones, with or without intrahepatic biliary strictures, ectatic and dilated intrahepatic ducts, liver atrophy, or liver abscesses, thirdly, confirmed pathological features of RPC on histological examination in patients who had undergone hepatectomies.

The exclusion criterion was defined as the presence of a neoplastic (i.e., cholangiocarcinoma), congenital (i.e., choledochal cysts or Caroli's disease), autoimmune (i.e., primary sclerosing cholangitis), or postoperative causes for the biliary obstruction.

### 3.1. Assessment of Severity and Extent of Disease

The definition of the extent of ductal involvement is in accordance with the terminology of the hepatic anatomy sanctioned by the International Hepatobiliary Pancreatic Association [11]. The complexity of the bile duct involvement was deemed to be simple if the disease was restricted to first order ducts and complex if the disease involved the second and third order ducts. 

Routine assessment of the bile ducts with intraoperative choledochoscopy was performed for those cases that underwent operative management. For cases which underwent the nonoperative treatment options (i.e., ERCP and PTC guided procedures), assessment of the ducts was based on the cholangiography images. Strictures were defined as a narrowing of the bile duct calibre in comparison with an undiseased bile duct of a similar generation associated with proximal dilatations of obstructed ductal system. Ectasia of the bile duct was determined by the presence of irreversible dilatation of the bile ducts despite an adequate drainage procedure with decompression of the obstructed system. Atrophy were determined if there was obvious atrophic changes of the liver parenchyma on the radiological findings or intraoperative assessment.

All patients underwent preoperative imaging of the biliary tract that included either computed tomogram (CT) or magnetic resonance imaging (MRI) assessment of the liver and extrahepatic biliary tree. Intraoperative assessment of liver abscess and atrophy was performed and confirmed with histology where biopsy or resection was performed. 

### 3.2. Initial Management of Acute Cholangitis Episode

The initial conservative measures for acute cholangitis consist of intravenous broad-spectrum antibiotics, fluids, adequate analgesia, and close observation of the clinical condition. In some patients when there are signs of severe cholangitis such as persistent fever, severe sepsis, septic shock, worsening abdominal tenderness, emergent therapeutic decompression, and drainage of the biliary tract is necessary. This can usually be achieved by endoscopic retrograde cholangiopancreatoography (ERCP) guided stent insertion or sphincterotmy and removal of obstructing calculi. This has the advantage of immediate biliary decompression if the site of obstruction is at the common bile duct (CBD). In situations where ERCP guided removal of calculus or stent placement drain is inadequate, an addition of a percutaneous transhepatic biliary decompression is necessary. After resolution of the acute episode of cholangitis, definitive treatment is planned. 

### 3.3. Definitive Management

**Figure 1:**
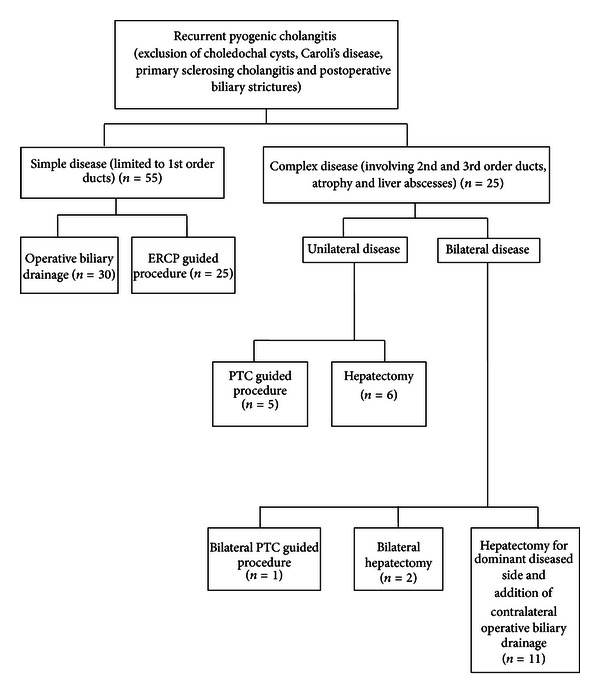
Management algorithm of recurrent pyogenic cholangitis.

For patients with isolated disease in the first order ducts, either operative biliary drainage or non-operative drainage by ERCP guided procedures was chosen based on surgeon decision and assessment of the patient's condition. For patients with stones or strictures in the second order ducts and beyond, PTC guided treatment or hepatectomy were offered. In cases where there were concomitant liver abscesses, or liver atrophy, hepatectomy was offered preferentially (see Figure 1).

### 3.4. Treatment Definitions

Operative management options are described as follows: operative biliary drainage procedure which includes exploration of the common bile duct and extraction of the calculi which was performed by stone forceps, balloon catheters, or dormia baskets. Strictures, if present, were mechanically dilated. If those strictures were deemed accessible surgically, they were resected. Biliary reconstruction was typically performed with a high choledochojejunostomy (above the level of cystic duct insertion) or hepaticojejunostomy. 

The definition of the hepatectomy was performed in accordance with the International Hepatobiliary Pancreatic Association definitions [11]. Hepatectomy was performed with the use of an ultrasonic surgical aspirator. Pringle's maneuver was applied to occlude the inflow of the liver when required. Each Pringle's occlusion was limited to 15-minute clamp time with 10-minute unclamped intervals. Operative biliary drainage procedure was added if the stones could not be cleared by hepatectomy alone, usually because of contralateral intrahepatic stones or impacted common hepatic/bile ducts stones. 

All patients who underwent operative management options underwent cholecystectomies when the gall bladder was not previously resected. All patients underwent intraoperative choledochoscopy to confirm the level of disease before the treatment and after the operative treatment to confirm clearance of the calculi or successful treatment of the biliary strictures if present. 

Non-operative management options are described as follows. Endoscopic retrograde cholangiopancreatography (ERCP) guided procedures include the use of ERCP for sphincterotomy, stone extraction with the dormia basket, and balloon catheter, with or without placement of a biliary stent. 

Percutaneous transhepatic cholangiography (PTC) guided procedures were employed when there was no liver atrophy or when patients refused surgery. PTC guided procedures included balloon catheter or dormia basket assisted removal of stones, stricture dilatation, and placement of biliary drains.

### 3.5. Follow-Up

Postoperative morbidity and mortality were defined as complications or death within 30 days of the operation. The effectiveness of each therapeutic modality was assessed for initial stone clearance immediately after the procedure and the development of recurrent cholangitis, recurrent stones, or liver abscesses during the follow-up period. Initial failure was defined as the presence of residual stones immediately after the procedure. Residual stones were confirmed by the cholangiography after the procedure was performed, when further removal of stones was not technically possible. Disease specific mortality was defined as the cause of death attributable to biliary disease, that is, cholangitis, liver abscesses, or liver failure. 

Long-term failure was defined as recurrent cholangitis, recurrent stones, or recurrent liver abscesses during the follow-up period. Recurrent cholangitis was diagnosed based on recurrent Bil >35, fever, and right hypochondrium pain. Recurrent liver abscesses were diagnosed based on ultrasound or CT imaging performed during the follow-up. The stone-free survival period and long-term mortality were also reviewed.

Statistical analysis was performed with SPSS version 17.0; Fisher's exact test was used to compare the proportions of treatment failure and complication rates between the groups. Logistic regression analysis was employed for multivariate analysis of the risk factors associated with treatment failure. The Kaplan Meier graph was used to express time to failure for the various treatment modalities. Statistical significance was defined as *P* < 0.05.

## 4. Results

### 4.1. Patient Characteristics

The patients' ages ranged from 22 to 95 years, with a mean age of 58.2 years. 31 were male and 49 were female. There were 48 Chinese, 22 Malay, two Filipino, two Bangladeshi, two Burmese, and four Vietnamese patients. 

The general characteristics of the patients were generally similar, except for the higher proportion of bedbound patients (*n* = 5, *P* = 0.007) in the group that underwent nonoperative management. With regard to the disease characteristics, the operative management group had a significantly higher proportion of patients with liver atrophy (*n* = 19,  *P* = 0.021); other differences in the disease characteristics did not reach statistical significance (see Table 1.).

Out of the 80 patients, 31 patients underwent nonoperative procedures. 25 of them underwent ERCP guided procedures and 6 of them underwent PTC guided procedures. Of the 49 patients who underwent surgical procedures, eight patients underwent hepatectomy only, 11 patients underwent hepatectomy combined with operative biliary drainage procedures, and 30 patients underwent operative biliary procedures only. Of those patients who underwent hepatectomy, two patients underwent bilateral hepatectomies for the treatment of bilateral disease. 

### 4.2. Outcomes of the Procedures

ERCP guided procedures had an initial failure rate of 36% (*n* = 9/25) and a 68% (*n* = 17/25) long term failure rate in terms of stone clearance. For the patients who underwent PTC guided procedures an initial failure rate was 16.7% (*n* = 1/6) and long term failure rate was 16.7% (*n* = 1/6). Overall, for non-operative treatment, the collective initial failure rate of stone clearance was 32.3% (*n* = 10/31), and the long-term failure rate was 58.1% (*n* = 18/31).

Of the patients who underwent operative biliary drainage only, three out of 30 patients had residual stones. Seven out of the 30 patients had recurrent cholangitis or stones on followup. Thus the initial failure rate was 10.0% (*n* = 3/30) and long-term failure rate was 23.3% (*n* = 7/30).

There were no residual stones or long-term treatment failures for the eight patients who underwent hepatectomy only. The two patients who underwent bilateral hepatectomy did not suffer from initial or long-term failure. For the patients who underwent combined hepatectomy and operative biliary drainage, the initial failure rate was 18.2% (*n* = 2/11) and long-term failure rate was 27.3% (*n* = 3/11), due to the residual stones in the third order ducts of the unresected contralateral liver and recurrent cholangitis and stone, respectively. Considering all forms of operative management, there were an initial failure rate of 10.2% (*n* = 5/49) and a long-term failure rate of 20.4% (*n* = 10/49).

Initial failure rate in terms of residual stone of operative compared with non-operative treatment was 10.2% versus 32.3% (*P* = 0.020). Long-term failure rate for operative compared with non-operative treatment was 20.4% versus 61.3% (*P* = 0.010) (see Table 2).

Based on multivariate logistic regression, the only significant factors associated with failure were bilaterality of disease (OR: 8.101, *P* = 0.007) and non-operative treatment (OR: 26.843, *P* = 0.001) (see Table 3).

**Figure 2:**
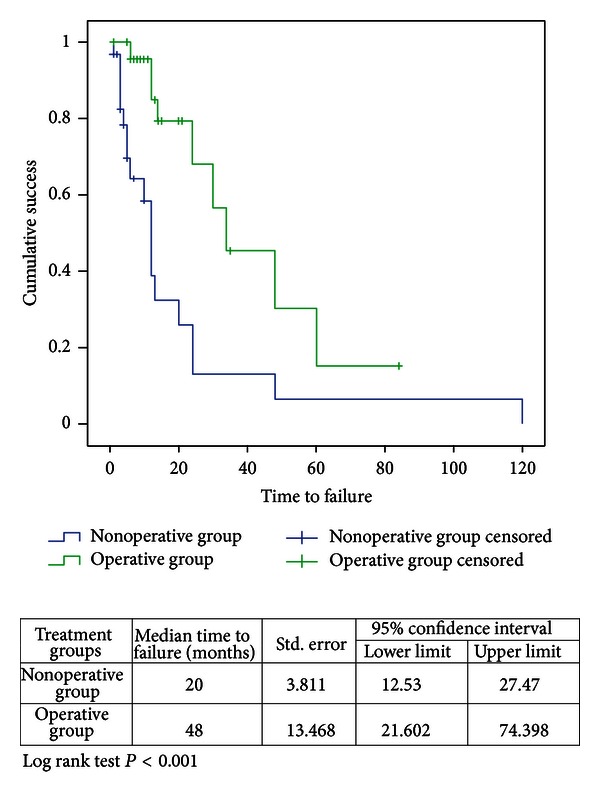
Kaplan Meier curve for comparing non-operative procedures versus operative procedures.

The median time to failure of the operative group was 48 months as compared to 20 months in the nonoperative group (*P* < 0.010). The duration of followup ranged from 10 to 120 months. There were no patients lost to followup, censored data was attributable to the fact that the event (failure) has not occurred yet (see Figure 2).

### 4.3. Postoperative Morbidity and Mortality

The general complication rate of the operative procedures was 44.9% versus 22.5% in the non-operative group (*P* = 0.057). The significantly surgical complications were mainly attributable to wound infection, wound dehiscence, and bleeding which was not observed in the non-operative treatment group (see Table 4).

The postoperative biliary complications rate for the nonoperative group was 16.2% versus 10.2% for the operative group (*P* = 0.513). Among the biliary complications, the rates for bile leak and biloma formation were 8.2% in the operative group versus 3.2% in the non operative group (*P* = 0.647); the rates of cholangitis in the perioperative period were 9.7% in the non-operative group versus 2.0% in the operative group. (*P* = 0.291). There was no initial or long-term failure observed in the two patients who underwent bilateral hepatectomy (see Table 5).

There was one mortality for the operative group due to postoperative myocardial infarction. There were four mortalities in the non-operative group, one patient passed away due to recurrent cholangitis and the other three passed away due to pneumonia and cerebrovascular accidents.

## 5. Discussion

Recurrent pyogenic cholangitis is a disease that is difficult to completely eradicate. This is because of the resultant strictures and ectatic bile ducts which predispose to bile stasis, formation of biliary calculi, and recurrent infection [6, 7]. Furthermore, the disease may result in atrophy of the affected liver segments and formation of abscesses as a consequence of the obstruction of biliary drainage and recurrent cholangitis. 

Thus the optimal treatment methods are directed at stone clearance and prevention of recurrent disease. This can be achieved by establishing adequate biliary drainage with or without removal of the diseased biliary tract by formal hepatic/biliary tree resection. This treatment option is fairly established in the setting where there is associated liver atrophy, liver abscesses, or third order ductal calculi [12–15]. However, there are many constraints for this method of treatment. Inadequate liver remnant, bilobar involvement, and poor patient fitness to tolerate major resection are several factors limiting liver resection as the treatment of choice. Furthermore, RPC involving the first order ducts is mainly treated by biliary drainage procedures via endoscopic, radiological, or surgical routes, without the need for hepatectomy. 

Based on our management approach, our methods of non-operative interventions, ERCP and PTC guided procedures, had a 58.1% failure rate and were significantly associated with treatment failure on multivariate analysis. This reflected a need for a better method of non-operative stone extraction and biliary dilatation. In our institution, ERCP guided procedures have been shown to have a high failure rate of 68%. This could be due to the failure of complete clearance of stones, most likely limited by the technical constraints of the procedure and the nature of the stones associated with RPC which are soft brown pigmented stones prone to leaving residue despite best efforts. 

PTC guided procedures mainly employed for access of second or third generation ducts have stone retrieval and stricture dilatation capabilities, which could have accounted for the lower failure rate as compared to ERCPs. Nonetheless, these non-operative methods have an important role as a temporising procedure in the management of patients presenting with acute cholangitis. As shown in our study, the median time of failure of 20 months would mean that this procedure can be employed as a first line treatment for the elderly patient with significant surgical risks and yet is durable enough. Based on the results, repeat ERCPs or PTCs should be planned within 20 months to reduce the risk of recurrent cholangitis. 

Surgical treatment of RPCs generally yielded better results than non-operative techniques due to the surgical removal or bypass of the diseased biliary tract along with the eradication of stones in those disease bile ducts. In our series, the low rate of failure from operative biliary drainage alone has been encouraging. Although other studies by Kusano et al. [16] and Li et al. [17] had high rates of recurrent stones and cholangitis, this was not observed at our institution. This could be attributable to the fact that we performed resection or dilatation of the associated biliary strictures where possible. In addition, the routine intraoperative choledocoscopy aided the identification of the location of the strictures and stones and subsequent confirmation of stone clearance. We routinely performed a high biliary bypass with the bile duct above the level of cystic duct insertion or at the level of the hepatic duct to maximise the size of the biliary conduit. There was high proportion of patients with biliary ectasia in our cohort which possibly contributed to the patency of the biliary drainage. 

There were two treatment failures in the combined operative biliary drainage and hepatectomy group due to the inability of complete stone clearance of the third order ductal stones in the contralateral hemiliver which was not resected. In the multivariate analysis, our data showed that patients with bilateral disease had a significantly higher risk of treatment failure. Similar observations were made by other studies, where bilateral disease often represented more complex disease and was treated aggressively. It has been reported in other papers that bilateral hepatectomy in which liver resection of all stone bearing segments in patients with bilateral disease achieved similar outcomes as surgical resection for patient with only unilateral disease [8, 15, 18]. In our series, only two patients have undergone bilateral hepatectomy for treatment of bilateral intrahepatic calculi and there was no recurrent disease seen in those patients. 

Considering all the treatment modalities, there was a higher rate of failure in the non-operative treatment group as compared to that of the operative treatment group. This was not withstanding the relatively less severe and extensive disease for those who underwent non-operative treatment modalities. This demonstrates that the optimal non-operative management option remains to be defined. Currently, there are many East Asian centres in Taiwan, Japan, and Korea with a high incidence of RPC, developing various methods of peroral or percutaneous cholangioscopic treatment modalities with encouraging results [9, 10, 19, 20]. Those methods have also become the main non-operative treatment modalities in preference to cholangiography guided options. 

It was also observed that the operative treatment group had a higher risk of developing complications of bile leak and biloma, although these too did not reach statistical significance. The reason for the bile leakge as explained by Li et al. [21] was related to unidentified aberrant biliary anatomy and recurrent cholangitis impairing bile duct healing. In addition, even bile leak tests performed intraoperatively cannot predict postoperative bile leak and biloma formation [22]. Despite these risks associated with operative procedures, the complication rate is relatively low and there were no mortalities related to biliary complications. 

The durability of the different therapeutic modalities for RPC is also a crucial determinant of the effectiveness of those options. From the Kaplan Meier curve, it can be observed that operative procedures are almost three times more durable than non-operative methods. Thus in an otherwise healthy patient, it is more effective to undergo an operative procedure albeit the slightly higher complication rate. Although less durable, non-operative treatment methods can still be employed as temporizing procedures or continued to be offered as definitive therapy for elderly patients with poor premorbid status and a shorter life expectancy. 

It is known that chronic proliferative cholangitis (CPC) initiated by stone mechanical stimulation or repeated acute cholangitis may persist and progress within the remaining bile ducts even after stone removal. CPC leads ongoing fibrotic thickening or restenosis of the bile ducts and hyperplasia of submucosal glands that hypersecrete mucoglycoprotein. Mucoglycoprotein, an important lithogenic factor, together with bile duct restenosis, facilitates stone recurrence [23]. Some authors have investigated the value of performing chemical biliary duct embolization (CBDE) to eradicate CPC and thereby prevent the recurrence of intrahepatic calculi. However, this technique has been restricted due to the cost of complete destruction of the subsidiary hepatic segment and related bile duct [24]. Hence, until newer techniques to arrest the process of CPC can be found, the best available therapy remains the current available techniques of ductal clearance.

The authors acknowledge the limitations of this study. Being a retrospective review that spans a 10-year period, it would have seen advances in diagnostics, perioperative care, and improvements in endoscopic and surgical techniques. A prospective controlled trial would be ideal to better define the optimal treatment modality and outcomes between the different therapeutic modalities available. However, the varied presentation and complication, relative paucity of cases, and wide variety of therapeutic options continue to pose a formidable challenge, given the evidence of other studies [5, 7, 12, 19].

Despite the limitations of the study, the results of this study suggest that with appropriate case selection, operative treatment would yield more favourable and durable results over non-operative methods for the treatment of RPC.

## Figures and Tables

**Table 1:** Comparison of demographics and disease characteristics between the operative versus non-operative groups.

	Operative group (*n* = 49)	Non-operative group (*n* = 31)	*P* value
Mean age	56.98	60.55	0.406 (NS)
Gender			
Male	19	12	0.995 (NS)
Female	30	19	
Race			
Chinese	29	19	0.425 (NS)
Non-Chinese	20	12	
Malay	14	8	
Vietnamese	4	0	
Burmese	1	1	
Filipino	1	1	
Bangladeshi	0	2	
Comorbidities (DM, HTN, IHD, hyperlipidemia, or CVA)			
≥3 comorbidities	13	7	0.691
<3 comorbidities	36	24	
Bedbound	0	5	0.007 (Fisher)
Non-bedbound	49	26	
Biochemical characteristics			
Albumin (mean)	31.90	32.48	0.646 (NS)
Bilirubin (mean)	58.71	72.37	0.296 (NS)
Disease characteristics			
Unilateral	34	16	0.110 (NS)
Bilateral	15	15	
Simple	37	26	0.149 (NS)
Complex	12	5	
Stricture			
No	36	22	0.807 (NS)
Yes	13	9	
Ectasia			
No	28	22	0.213 (NS)
Yes	21	9	
Liver atrophy			
No	30	27	0.021
Yes	19	4	
Liver abscess			
No	37	29	0.067
Yes	12	2	

**Table 2:** Comparison of the initial and long-term failures of different treatment modalities.

Treatment modality	Initial failure	Long-term failure
ERCP guided	9/25 (36.0%)	17/25 (68.0%)
PTC guided	1/6 (16.7%)	1/6 (16.7%)
Non-operative treatment	10/31 (32.2%)*	18/31 (58.1%)^†^
Operative biliary drainage only	3/30 (10.0%)	7/30 (23.3%)
Hepatectomy only	0/8	0/8
Hepatectomy and operative biliary drainage	2/11 (18.2%)	3/11 (27.3%)
Operative treatment	5/49 (10.2%)*	10/49 (20.4%)^†^

*P = 0.020, ^†^ *P* < 0.010; Fischer's exact test.

**Table 3:** Multivariate logistic regression analysis of factors associated with long-term failure.

Factor	Adjusted OR	95% CI	*P* value
Age	1.032	0.991–1.075	0.129 (NS)
Gender			
Female	1		
Male	0.341	0.083–1.402	0.136 (NS)
Bedbound			
No	1		
Yes	7.332	0.419–128.341	0.173 (NS)
Albumin	0.886	0.774–1.014	0.078 (NS)
Bilirubin	0.996	0.984–1.008	0.522 (NS)
Laterality			
Unilateral	1		
Bilateral	8.101	1.766–37.156	0.007
Complexity			
Simple	1		
Complex	2.050	0.304–13.809	0.555 (NS)
Stricture			
No	1		
Yes	2.564	0.530–12.403	0.242 (NS)
Ectasia			
No	1		
Yes	0.895	0.184–4.355	0.891 (NS)
Liver atrophy			
No	1		
Yes	3.584	0.594–21.619	0.164 (NS)
Liver abscess			
No	1		
Yes	6.058	0.960–38.230	0.055 (NS)
Extrahepatic stones			
No	1		
Yes	1.228	0.274–5.509	0.789 (NS)
Treatment			
Operative	1		
Non-operative	26.843	4.856–148.394	0.001

**Table 4:** Postoperative general complication for all procedures.

Procedure	Complications					
	Pneumonia	UTI	Wound infection	Wound dehiscence	Bleeding	Total
ERCP	2	3	0	0	0	5/25
PTC	1	1	0	0	0	2/6
Non-operative	3	4	0	0	0	7/31
Operative biliary drainage only	2	4	4	3	0	13/30
Hepatectomy only	1	1	1	1	0	4/8
Combined hepatectomy and operative biliary drainage	0	1	2	1	1	5/11
Operative	3	6	7	5	1	22/49

**Table 5:** Postoperative biliary complications for all procedures.

Procedure	Complications			
	Cholangitis	Bile leak and biloma	Hemobilia	Total
ERCP	3	0	0	3/25
PTC	0	1	1	2/6
Non-operative	3/31	1/31	1/31	5/31
Operative biliary drainage only	1	1	0	2/30
Hepatectomy only	0	2	0	2/8
Combined hepatectomy and operative Biliary drainage	0	1	0	1/11
Operative	1/49	4/49	0/49	5/49
